# Dr. William J. Hennessy

**Published:** 1918-07

**Authors:** 


**Dr. William J. Hennessy** of Palmyra died April 27, age 61. He graduated from the Syracuse University Medical College in 1881.

